# The hope wheel: a model to enable hope-based pedagogy in Climate Change Education

**DOI:** 10.3389/fpsyg.2024.1347392

**Published:** 2024-03-20

**Authors:** William Finnegan, Cathy d’Abreu

**Affiliations:** ^1^Department of Education, University of Oxford, Oxford, United Kingdom; ^2^Oxford Brookes Business School, Oxford Brookes University, Oxford, United Kingdom

**Keywords:** climate change, education, pedagogy, hope, education for sustainability

## Abstract

In response to concerns about climate anxiety and distress, researchers and practitioners in both education and psychology have been investigating the importance of engaging climate hope in Climate Change Education (CCE). Synthesizing recent multidisciplinary research, alongside insights from the development of educational programs, this article proposes a new theoretical model for pedagogies of hope in CCE. The Hope Wheel presents three foundational elements: handrails for educators to hold on to while constructively engaging with climate change (honesty, awareness, spaceholding, action), guardrails for educators to be sensitive to when implementing the handrails (climate anxiety, mis-/disinformation, false hope), and lenses to encourage educators to explore connections between complex societal and planetary challenges (complexity, justice, perspectives, creativity, and empathy). This working model aims to support educators by distilling current learnings from the literature into a visual guide. It depicts essential elements to include, as well as avoid, in order to engage honest, hope-oriented CCE for transformative learning in the face of the climate crisis.

## 1 Introduction

Educators increasingly acknowledge the importance of engaging with climate change across a broad range of subject areas and its current relevance from both a pupil and planetary perspective (Edge Research, 2022; Teach the Future, 2022). There are, however, a number of commonplace barriers, compounded by a lack of adequate resources, that problematize embedding climate education in classrooms, presenting considerable challenges for both teachers and learners alike to navigate (Howard-Jones et al., 2021; Greer et al., 2023).

Both the scientific and emotional aspects of climate education need not only evidence-based approaches that are theory-informed, but also require readily navigable, digestible signposting for busy teachers with limited training and capacity. The literature points to overcrowded curricula, ideological considerations, and a lack of expertise and development opportunities that can result in limited confidence to take up the challenges involved in the complex, multidisciplinary nature of CCE efforts (Rousell and Cutter-Mackenzie-Knowles, 2020).

In response to concerns about climate anxiety and distress, many researchers and practitioners in both education and psychology have increasingly acknowledged the need for hope-based approaches, the most prominent of which are those that headline constructive, active, critical and transformative ideas of hope-based learning (Ojala, 2012; Kerret et al., 2016; Li and Monroe, 2019). Whilst the theoretical framework around the importance of hope in CCE gains traction, operationalizing these concepts can feel both daunting and abstract for educators faced with the practical realities of including climate education in everyday teaching and learning settings.

The evidence-based working model proposed in this Curriculum, Instruction, and Pedagogy (CIP) article aims to bridge the gap between research and practice around how to constructively cultivate hope in the face of the climate crisis with learners of all ages, as well as encourage educator confidence in starting explorative discussions without the expectation of discrete, concrete solutions. We aspire to simplicity and accessibility in the model, while recognizing the complexities and challenges of engaging with a concept like climate hope. The Hope Wheel aims to support the process of transformative learning for the social transformations needed that will necessarily involve moments of discomfort and challenge for both educators and learners (Mezirow and Taylor, 2009).

In response to the complexity of these educational challenges, the Hope Wheel model offers a visual synthesis of foundational “handrails,” “guardrails,” and “lenses” for constructively engaging with climate change across a broad range of subjects and disciplines, thereby facilitating hope-oriented pedagogy for CCE.

## 2 Background

This article builds on a wide range of scholarship in environmental, sustainability and climate education, as well as relevant theory in environmental/educational psychology and transformative education.

### 2.1 Climate Change Education (CCE)

As anthropogenic climate change has been acknowledged as an existential threat to human and natural systems (IPCC, 2023), education has been affirmed as a key vector for driving the behavior change necessary for the paradigm change and social transformation needed (Otto et al., 2020; United Nations., 2021). Article 14 of the Paris Agreement of the United Nations Framework Convention on Climate Change called for all parties to enhance CCE as a means of limiting global heating to 1.5 degrees Celsius (United Nations, 2015a). This is further bolstered by Sustainable Development Goal (SDG) 4.7, which states that by 2030, governments must “ensure that all learners acquire the knowledge and skills needed to promote sustainable development” (United Nations, 2015b).

Climate Change Education has emerged from a wide range of established educational fields including environmental education (EE), Global Citizenship Education (GCE), Education for Sustainability (EfS) and Education for Sustainable Development (ESD) (Bourn and Hatley, 2022), with a particular emphasis placed on engaging with and envisioning probable, possible and preferable futures (Kagawa and Selby, 2010; Hicks, 2014). In a sweeping review of CCE, Reid (2019) documented key trajectories of CCE practice and research, across the cognitive, social-emotional and behavioral dimensions, highlighting the need to engage with “hard truths” of climate change, alongside enabling action to participate in mitigation and adaptation efforts. Reid articulates the challenge for CCE to move beyond climate science literacy to activating “response-ability” (Sterling and Martin, 2019). This requires a “shift in our lifestyles and a transformation of the way we think and act. To achieve this change, we need new skills, values and attitudes that lead to more sustainable societies” (Reid, 2019).

In a systematic review of research evaluating CCE practices, Monroe et al. (2019) identified several essential strategies in CCE, including engaging in deliberative discussions, interacting with scientists, addressing misconceptions, and implementing school or community projects. Headlining the need to address misconceptions is particularly important in countries where climate change is highly politicized and misinformation rampant (Government Office for Science, 2023). Critical thinking skills, as described in the UNESCO (2017) sustainability competencies, are one means of addressing misinformation, as are developing digital, data and information literacies (Organization for Economic Co-operation and Development [OECD], 2018).

Also integral to CCE is the relevance of emotions, as reflected in the “bicycle model” of CCE proposed by Cantell et al. (2019) which included an element related to hope and other emotions. The pivotal role of emotions and their impacts in the classroom was further explored by Verlie (2019), who shifted the role of CCE from “preventing or fixing” to “learning to live” with climate change, a process of acknowledging affects such as loss, anger and grief. This “bearing worlds” is presented as a requirement of climate adaptation, moving CCE toward fostering the knowledge, skills and capacities to navigate change and uncertainty and, importantly, foster empathy for the self and others within this difficult landscape. Here hard truths and hope are explicitly linked to change:

*Learning to live-with climate change is therefore a process of bearing worlds, as we simultaneously become more attuned to our enmeshment with the more-than-human, mourn those relationships as they are ruptured, act-with them to cultivate the most promising futures possible, and are ourselves changed throughout the process (2019, 752).*

### 2.2 Climate hope

The concept of hope has a rich, contested and complex history in philosophy, theology and psychology, with Webb (2013) distinguishing five modes of hoping in his review of pedagogies of hope: patient hope, critical hope, sound hope, resolute hope and transformative hope. Pedagogies of hope have also been developed by critical educational theorists such as Freire (2004) and hooks (2003), who connect hope with individual transformation: “To successfully do the work of unlearning domination, a democratic educator has to cultivate a spirit of hopefulness about the capacity of individuals to change” (hooks, 2003, 73).

The field of positive psychology has contributed further understanding through Snyder’s hope theory, which identifies the core hope drivers as goal identification, pathways thinking (waypower), and agency thinking (willpower) (2002).

As a cognitive process connected to both emotional states and behavior, hope is particularly relevant to how educators engage with climate change. Ojala’s exploration of climate and hope, including the factors “trust in self” and “trust in others,” concluded that “constructive hope” is central to environmental engagement in young people (Ojala, 2012, 635). This echoes the writings of Macy and Johnstone (2022) on Active Hope, in which they provide a relevant and accessible definition:

*Active Hope is a practice. Like tai chi or gardening, it is something we do rather than have. It is a process we can apply to any situation, and it involves three key steps. First, we start from where we are by taking a clear view of reality, acknowledging what we see and how we feel. Second, we identify what we hope for in terms of the direction we’d like things to move in or the values we’d like to see expressed. And third, we take steps to move ourselves or our situation in that direction (2022, 4–5).*

Research has applied constructive hope to secondary school climate education, including work confirming the relationship between hope and action competence (Ojala, 2015; Li and Monroe, 2018; Finnegan, 2022). Finnegan (2023) also explored creative pedagogies–digital storytelling about climate futures–as a means of encouraging hope through positive reappraisal, a cognitive process to support coping and adaptation in which something perceived as negative is re-evaluated and personally meaningful positive steps are identified.

There is also growing interest among researchers in the broad range of emotional responses to climate change and the corresponding interrelationships between wellbeing, learning and action. This includes the importance of acknowledging the impacts of climate anxiety and distress (Clayton, 2020; Hickman et al., 2021), as well as recognizing the broad range of climate emotions (Pihkala, 2022). Ojala (2021) connects these broader affective elements to the concepts of pedagogies of discomfort (Boler and Zembylas, 2002) and critical emotional awareness (Ojala, 2023), noting their importance in designing and delivering effective CCE. At the same time, both the intensity of emotional responses to climate change and the very real psychological impacts of traumatic climate change experiences can give educators pause (Clayton et al., 2023).

The critique of false hope has been made by Bendell (2023) and others in the deep adaptation movement. In these circles, those that self-identify as “doomsters” dismiss political and technological climate solutions as “hopium” (Doig, 2023). Instead, they believe that the collapse of civilization is inevitable, with responses ranging from apocalypse prepping to permaculture (Bromwich, 2020). In many ways, the critique of false hope is more related to patient hope (Webb, 2013), hope based in denial (Ojala, 2012), and the misrepresentation of hope as optimism, which educational philosopher Dewey (2008) rejected as encouraging: “a fatalistic contentment with things as they are” (2008, 294). Further distinguishing between hope and optimism, Orr (2007) commented:

*Optimism is the recognition that the odds are in your favor; hope is the faith that things will work out whatever the odds. Hope is a verb with its sleeves rolled up. Hopeful people are actively engaged in defying or changing the odds. Optimism leans back, puts its feet up, and wears a confident look knowing that the deck is stacked (2007, 1392).*

### 2.3 Transformative learning

Many of the aforementioned tenets of hope-based learning are supported by the literature on Transformative Learning (TL) theory (Mezirow, 2000; Taylor and Cranton, 2012), which underpins the learner-centered, action-oriented, relational approaches to CCE offered by EfS and ESD. These pedagogies champion a holistic and transformational education which “addresses learning content and outcomes, pedagogy and the learning environment… and achieves its purpose by transforming society” (QAA and Advance HE, 2021). The TL process necessarily begins with self-awareness, facilitating change “from the inside out” (d’Abreu and Cripps, 2023) and centers on developing socially and environmentally critical thinking that challenges unsustainable normative behaviors and importantly, “empowers learners to take informed decisions and responsible actions for environmental integrity, economic viability and a just society” (UNESCO, 2017).

Mezirow’s definition of TL highlights this agentive purpose as being both an individual and collective endeavor describing it as:

The process by which we transform our taken-for-granted frames of reference (meaning perspectives, habits of minds, mind sets) to make them more inclusive, discriminating, open, emotionally capable of change, and reflective so that they may generate beliefs and opinions that will prove more true or justified to guide action. Transformative Learning involves participation in constructive discourse to use the experience of others to assess reasons justifying these assumptions, and making an action decision based on the resulting insight (Mezirow, 2000: 8).

Mezirow highlights that TL is triggered by “disorienting dilemmas,” highly pertinent in the CE context. A liminality state characterizes transformation from the safety of “established frames of reference” to new experiences or understandings that often involve loss, uncertainty and discomfort (Taylor and Cranton, 2012), mirroring calls to acknowledge and engage with the affective elements of CCE outlined above. Core to TL is engaging with this discomfort, sitting with uncertainty and creating action pathways by consciously “moving from safe to brave spaces” (Winks, 2018). Also critical is the development from individual to collective engagement, multiple perspective taking and relational learning with others and other discipline areas. Drawing on a range of current, relevant multidisciplinary sources that intersect with TL pedagogy, the Hope Wheel aims to enable TL in the CCE context that responds to the complexity and challenges educators and learners face.

## 3 Pedagogies of hope

The Hope Wheel was designed to bridge the gaps between theory in educational psychology research and classroom practice by translating key elements supporting a climate hope approach into guidelines. The model includes core climate hope concepts organized into the following digestible categories: handrails, guardrails and lenses to guide educator engagement with the challenge and complexity of CCE.

### 3.1 Methodology

The Hope Wheel is the result of the authors review of the literature, as captured in the Background section above, and their experiences in designing and delivering CCE, which are explored in the Discussion section that follows. As a Curriculum, Instruction and Pedagogy (CIP) article, this model does not represent a systematic review, nor is it the result of longitudinal or experimental studies. Rather, the Hope Wheel reflects a timely synthesis of theory, empirical studies and experience into what the authors intend to be a practical tool for both educators and researchers.

### 3.2 Handrails

The first handrail highlights the crucial importance of Honesty and telling “hard truths” about climate situations, (contrasting with the mis-/disinformation and false hope guardrails later explored). It signposts that transformative CCE is not just about delivering, deciphering or digesting accurate scientific facts, but needs to be coupled with a solutions-orientated approach to enable hope-based solutions.

The Awareness handrail highlights that self-reflection is core to the transformative learning process and is initiated by developing critical awareness of the nested interconnections linking the self, others and the more-than-human world. Here identifying the relational, situated realities of climate leaning and embedded emotions are important, as is the ability to question normative narratives that present biased, inaccurate or exclusive understandings of climate change dilemmas (Wals, 2007).

The Spaceholding handrail acknowledges that enabling both safe and brave spaces is crucial to protect leaners and the emotional engagement CCE involves, while also empowering their potential agency. Here again, we engage with an important tension–holding a space for emotional reflection and transformation, while deflecting denial, disengagement or disempowerment by creating “safe-enough” spaces for constructive hope in CCE to flourish (Weintrobe, 2021; Hamilton, 2022; Singer-Brodowski et al., 2022).

Both awareness and spaceholding can support emotional engagement with climate change, critical to empowering hope-based, transformative, action-oriented learning opportunities.

Lastly, the Action handrail headlines moving from merely problematizing issues to creating purposeful pathways through motivating both individual and collective action. Important here is the recognition that individual actions, though essential, need to be scaled up and supported through collective efforts, both to avoid imposing unreasonable burden on learners and to enable both “willpower” and “waypower” (Snyder, 2002). Reflection on individual action and agency must be coupled with recognition that transformative change is a collective endeavor, a lifelong learning process in which we need to collaborate with others for success.

In the Hope Wheel, the handrails are represented as spokes of the wheel, illustrating the integrated nature of the elements in terms of the structural integrity of the wheel. In addition, each spoke highlights two aspects of the handrail which can be understood as in productive tension, which is further explored in Table 1.

**TABLE 1:** Hope Wheel spokes/handrails and educator practices (from-to).

*From*		To
*Absence*	*Imbalance*	
midrule Lack of honesty about the situation and solutions (climate silence)	Honesty about the problem of climate change without acknowledging the existence of solutions (doomism), or honesty about the solutions without fully acknowledging the seriousness of the situation (techno-optimism)	Honesty about both the situation and solutions
Lack of awareness about self and the world (unaware)	Self-aware but not aware of relationships/dependencies on others and the complex systems of the wider world (limited external awareness), or aware of others/world but personal motivations/strengths remain unexamined (limited internal awareness)	Awareness of self and world
Lack of spaceholding that is either safe or brave (no space)	Holding space that is safe but not brave (overcautious), or holding space that is brave but not safe (reckless)	Spaceholding that is safe and brave
Lack of engagement with either individual or collective action (inaction)	Focusing on individual actions, such as personal carbon footprints, rather than collective actions (individualism), or focusing on collective, systems-level action without engaging at a personal level (perceived hypocrisy)	Action that is both individual and collective

### 3.3 Guardrails

The guardrails of this model provide the outlines or rim to the Hope Wheel. They aim to guide engagement with the scientific and emotional dimensions of climate education by raising awareness of both what to acknowledge and what to avoid in the classroom.

The Climate anxiety guardrail acknowledges the need for educators to support learner wellbeing and avoid harm while exploring complexity, uncertainty and challenge in CCE issues and the range of emotions this encompasses. To avoid the triggers of climate distress and denial the Hope Wheel draws on trauma-informed practices to safeguard learner wellbeing. Educational psychology establishes the need to create ground rules such as being sensitive to learner lived experiences, giving clear trigger warnings, wellbeing breaks, time out and to make post-session one-to-one support available to learners (Singer-Brodowski et al., 2022). Also critical is to acknowledge and validate the broad range of climate emotions that may surface in a supportive, sensitive and non-judgmental fashion. Enabling and validating emotional engagement is critical to support learning of undoubtedly challenging themes.

The False hope guardrail warns against simplifying or sugarcoating issues in hope-based pedagogy. This supports the honesty handrail by ensuring that solutions and responses are not overly optimistic, simplified or unrealistic and that the distinctions between optimism and hope are examined critically. This guardrail also invites critique of doomist and techno-fix narratives that present disempowering or disingenuous conceptualizations of hope. Also germane to this guardrail is avoiding the pitfall of outsourcing hope by laying the burden of responsibility on individual learners (see more in the Discussion).

The Mis-/Disinformation guardrail highlights the necessity of developing digital, data and research literacy skills to ensure learners can identify false/misleading information, critically evaluate underlying biases and identify robust, reliable and trustworthy sources. This involves actively addressing misconceptions to prevent the spread of misinformation and build critical thinking capacity. This guardrail is related to the honesty handrail, as Frumkin (2022) noted, “Telling the truth means recognizing, naming – and countering the uncomfortable reality of deliberate disinformation promoted by vested interests” (2022, 4). It is also supported by the perspectives lens below that headlines the need for multidisciplinary approaches and acknowledging multiple worldviews.

### 3.4 Lenses

Additional to the core handrail and guardrail elements are the lenses to enable a holistic, equitable and inclusive understanding of CCE issues. Through these lenses, important cross-cutting themes related to all components of the Hope Wheel are made visible, ensuring a holistic range of critical viewpoints.

The Complexity lens acknowledges the absence of simple, linear, discreet solutions to climate change and notes the need for educators to lean into uncertainty, ambiguity and inherent contradictions. Highlighting that there are no “silver bullet” answers, either scientifically or emotionally, is challenging but essential if we are to respond honestly and authentically to students concerns and needs. Enabling a holistic, systems-thinking perspective (UNESCO, 2017) that explores the interconnected, interdependent tensions in CCE is therefore essential. This encourages a “birds eye view” of issues that considers the relational, contextualized and nested nature of global challenges.

The Justice lens ensures a historical perspective is included, inviting the question: How did we get here? It recognizes the impacts of colonialism and unequal global power structures and the extractivism they unleashed, in both human and environmental terms. This lens ensures the causes, impacts and proposed solutions for climate change are always critiqued from a social justice vantage point, making visible historic social, economic and environmental injustices and how their impacts today, and in the future, are disproportionately and unjustly distributed across social and geographical domains.

The Perspectives lens invites reflection and dialogue on how diverse individuals/communities/geographical locations perceive climate change issues and why. Learning from multiple perspectives and voices is paramount to enabling equitable solution pathways and understanding that climate change impacts are situated and contextual. This includes drawing from a range of subjects, discipline areas and multiple knowledges, and can be achieved by facilitating collaborations. Inclusivity and diversity are engaged through this lens.

The Creativity lens enables “What if…?” thinking opportunities while exploring the potency of creative solutions. Engaging creativity is a hopeful act in and of itself and can access and channel emotional responses toward positive solutions. Visioning is central to this lens, inviting students to envision probable, possible and preferable future scenarios driven by creativity. Encouraging and creating visions for the future–particularly positive, collective visions–underpins hope-based pedagogy, while also allowing expression of, and responses to, a range of climate emotions such as anger, joy and pain.

Lastly, the Empathy lens advances a culture of care, applying care, kindness and empathy to all aspects of this model, to ourselves, to others and to the more-than-human world in response to a “culture of uncare” (Weintrobe, 2021). Reflecting on one’s own and others’ feelings and emotions is encouraged and validated, as is developing knowledge, understanding and care of the natural world.

These lenses therefore are not presented as optional but rather integral to CCE, presenting essential layers through which to engage a holistic pedagogy that is critical, relational and emancipatory (Wals et al., 2009) drawing on multidisciplinary theoretical perspectives and evidence-informed practices.

**FIGURE 1:**
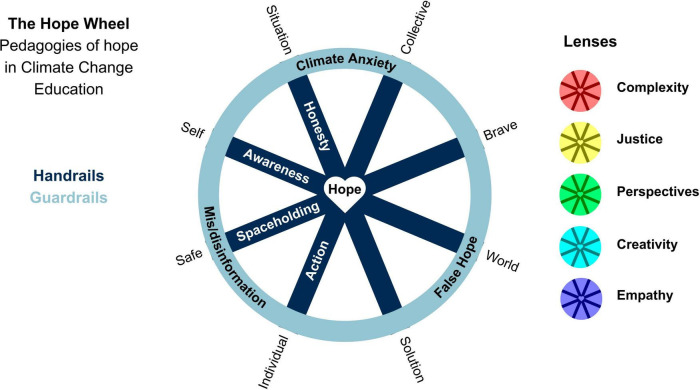
The Hope Wheel: handrails (spokes), guardrails (rim) and lenses to enable hope-based pedagogy in Climate Change Education.

Figure 1 illustrates the key elements of this model in an abstract manner through the visual metaphor of a wheel in which the handrails are spokes, the guardrails are the rim, and the lenses can be layered on top of the wheel. Table 2 presents each element in the model, questions related to this element that capture the pedagogical approach, and examples and resources for educators.

**TABLE 2:** The Hope Wheel: Applying the elements.

*Element*	*Pedagogical approach*	*Development areas and resources*
Honesty (about situation and solutions)	Is honest/truthful information presented? Are students enabled to explore/envision a range of (un)viable/possible actions and solutions?	Reliable sources for climate science, climate impacts, and climate solutions^3^
Awareness (of self and others/world)	Have students had the opportunity to reflect on: How they feel? What they fear? What their hopes are? How they do/can contribute to the issue? Are there opportunities to listen to and discuss issues with others?	Raising self-awareness, social awareness, connectedness, and environmental awareness^4^
Spaceholding (safe and brave)	Are safe spaces created that protect learner wellbeing and learner needs? Are there brave spaces created for discussing the realities, emotions and potential responses to CCE? Is a non-judgmental, inclusive and supportive environment enabled?	Create and maintain a safe and brave learning environment for handling complex issues inside and outside the classroom^5^
Action (individual and collective)	Are students aware of/able to explore their own agency and individual actions? Are collaborative opportunities for problem solving and local action explored, working outward from the classroom, school and wider community? Are examples of individual and collective action role models shared?	Whole school approaches to sustainability and action learning projects^6^
Climate anxiety (avoiding harm)	Sensitivity to learner welfare. Do any students come from frontline communities in terms of climate impacts? Have safeguarding ground rules — e.g., trigger warnings, wellbeing breaks, time out and post-session support — been established? Are all emotional responses validated and supported?	Develop ground rules for creating safe spaces while engaging with complex issues^7^
False hope (passive optimism)	Are issues sugarcoated? Are distinctions between hope and optimism explored? Are solutions/pathways presented as: A form of techno-optimism, in which individuals have limited agency? Over-optimistic, unrealistic, oversimplified, linear, binary or concrete?	Reading about hope, especially how the concept is expressed in literature and art, and exploring examples of hopeful constructive actions and those that are not^8^
Mis-/Disinformation (addressing misconceptions)	Are sources of information trustworthy, reliable and robust? How can false/misleading information be identified? What digital, research and data literacies can be developed? Are polarizations identified and critiqued?	Information and digital literacy, including skills related to evaluating sources of data for reliability and exposing misinformation^9^
Complexity	Are complexity, ambiguity, uncertainty and “no silver bullet” framings acknowledged? Is a holistic, systems thinking perspective enabled, looking at the interconnected issues in CCE? Is a bird’s eye view invited?	Developing systems thinking skills, for example with respect to the United Nations Sustainable Development Goals^10^
Justice	Are historical elements explored? ‘How did we get here?’ Are the disproportionate, unjust impacts of climate change explained? Are the causes, impacts and proposed solutions for climate change critiqued from a social justice vantage point? Whose voices have not been heard or are disadvantaged? Why?	Support learners to revisit assumptions, worldviews and power relations especially through exploring the experiences of climate change by frontline communities^11^
Perspectives	How do different individuals/communities/locations perceive climate change issues and why? Which school subjects/academic disciplines, are relevant? How can collaboration with others in an institution/community and beyond subject or discipline areas be enabled?	Sharing multiple perspectives, including through the arts and humanities^12^
Creativity	Can students envision probable, possible and preferable future scenarios? Enabling ‘What if…? thinking opportunities and exploring creative solutions. Is creativity validated as part of the change process? Is the process of creating visions for the future, particularly positive, collective visions, encouraged?	Digital storytelling projects and curating examples of creative solutions to climate change^13^
Empathy	Can students reflect on their and others’ feelings and emotions and develop their emotional connection to the natural world? Is applying care, kindness and empathy to all aspects of this model – to ourselves, to others, to the more-than-human world – supported?	Empathy exploration exercises^14^

^3^ For example, the Office for Climate Education’s summary for teachers of the IPCC’s 6th Assessment Report https://www.oce.global/en/resources/climate-science/ipcc-sixth-assessment-report-summary-teachers and Project Drawdown https://drawdown.org/drawdown-foundations.

^4^ For example, the Inner Development Goals https://www.innerdevelopmentgoals.org/resources and Thoughtbox Education’s triple wellbeing in the classroom model https://www.thoughtboxeducation.com/triple-wellbeing

^5^ For example, Force of Nature Discussion Guide for Educators https://www.forceofnature.xyz/discussion-guide and Climate Psychology Alliance Youth Support Space https://climatepsychologyalliance.org/index.php/component/content/article/youth-support-programme?catid=14&Itemid=101

^6^ For example, Sustainability and Environmental Education (SEEd) Whole Institution Approach to Sustainability https://se-ed.org.uk/our-work/whole-institution-school-approach-sustainability/

^7^ For example, PSHE Association’s resources for handling complex issues safely in the classroom https://pshe-association.org.uk/guidance/ks1-5/handling-complex-issues-safely-classroom

^8^ For example, the resources for educators from the All We Can Save Project at https://www.allwecansave.earth/for-educators

^9^ For example, Climate Action Against Disinformation’s climate mis-/disinformation backgrounder at https://caad.info/analysis/briefings/climate-mis-disinformation-backgrounder/

^10^ For example, the Donella Meadows Project https://donellameadows.org/systems-thinking-resources/and the Stockholm Resilience Center’s SDG wedding cake https://www.stockholmresilience.org/research/research-news/2016-06-14-the-sdgs-wedding-cake.html

^11^ For example, resources from Manchester Metropolitan University related to the HEADS UP model at https://www.mmu.ac.uk/research/projects/teaching-sustainable-development

^12^ For example, the Museum of Climate Hope https://climatehope.uk/and resources related to intercultural competencies and story circles at https://unesdoc.unesco.org/ark:/48223/pf0000370336

^13^ For example, the writings of Rob Hopkins, founder of the international Transition Towns movement https://www.robhopkins.net/the-book/

^14^ For example, the Rounder Sense of Purpose framework developed by University of Gloucestershire https://aroundersenseofpurpose.eu/framework/ec-inv/

**FIGURE 2:**
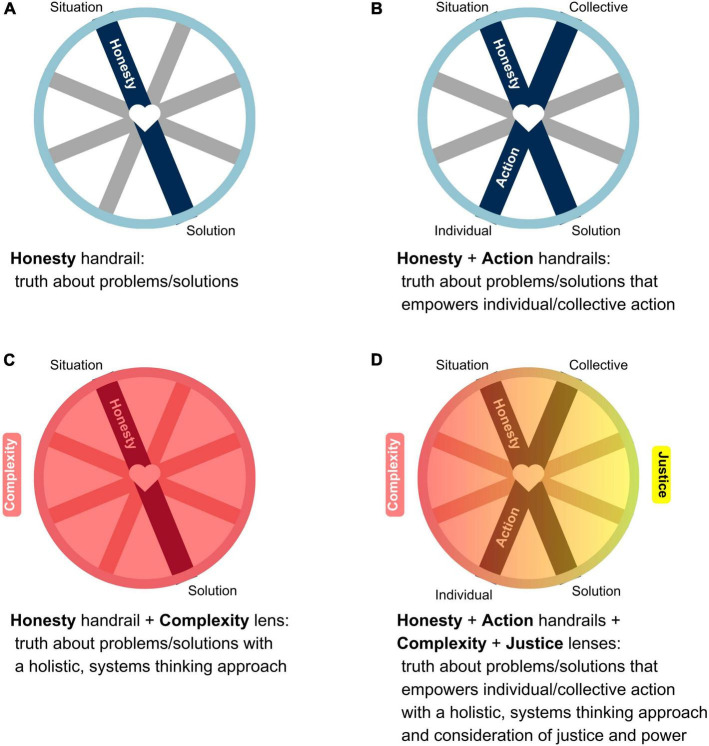
The application of the Hope Wheel: an example illustrating **(A)** the Honesty handrail, **(B)** the relationships between the Honesty and Action handrails, **(C)** the layering of the Complexity lens over the Honesty handrail, and **(D)** the layering of both the Complexity and Justice lenses over the relationship between the Honesty and Action handrails.

To further illustrate the application of the Hope Wheel, Figure 2 indicates how the elements interact – for example, how two handrails relate to one another, and how one or more lenses can be layered upon the handrails–in terms of how an educator might approach program design and evaluation.

## 4 Discussion

The Hope Wheel uses accessible, visual metaphors to provide guidance for educators in response to calls for bridging the gap between research and teaching for sustainability (Leal Filho et al., 2023). With such a model, even when synthesizing and responding to a broad range of literature, there are subjective decisions on what to include and how to design educational activities for educators. This article also reflects the choices of developing a working model based on a visual metaphor, and it is worth noting that a compass and flower were also considered as a means of illustrating the relationships between the various elements. We welcome other researchers and practitioners to continue to refine and improve this model and metaphor.

By aggregating central current principles from diverse disciplines, The Hope Wheel identifies pedagogical priorities for CCE that can “challenge students to participate actively, think critically and reflect” (Scarff Seatter and Ceulemans, 2017, 47). It champions challenging society’s dominant narratives and supports “transgressing the hidden curriculum of unsustainability: toward a relational pedagogy of hope” (Wals et al., 2009).

This process, Wals et al. go on to explain, encompasses three elements; a critical element, enabling the “space to ask bold and disruptive questions about why things are the way they are, to learn how things can be changed but also what keeps them from changing,” as well as exploring dis-/misinformation. A relational element, that connects the personal, inner self with other humans (“those not in sight, those thinking differently”) and the non-human world. Lastly, an emancipatory element that foregrounds agency through “autonomy and self-determination.” The concepts behind a pedagogy of hope are grounded in these tenets and encourage educators to intentionally design opportunities for “transgressive learning” to stimulate a shift in the way the learner sees the world.

Further to this, the Hope Wheel acknowledges the necessity to respond to young people’s well-documented concerns and anxiety around the climate crisis. We argue that this can be done through sensitive, hope-based, action-oriented approaches that protect learner wellbeing while empowering agency through creativity, collective action and a culture of care.

This article represents the first articulation of a theoretical conceptual model, and the authors acknowledge the need for rigorous testing and evaluation. Some current examples of operationalizing this model in practice are shared below, followed by a critique of climate hope.

### 4.1 Pedagogies of hope in practice

#### 4.1.1 Spaces, honesty and awareness handrails operationalized

Finnegan (2023) used speculative digital storytelling as both an educational intervention and participatory research method. This process–in which secondary school students participated in a series of workshops and produced video “letters from the future”–illustrates many aspects of the pedagogies of hope model. The workshops provided a “safe enough space” for emotionally engaging with climate change, in which difficult discussions were facilitated and honest information about the causes and solutions to climate change were presented. As a shared experience of reflection and support, the workshops also developed self-awareness and world awareness. Finally, the invitation to create a letter from their future self in the year 2050 supported both envisioning and communicating students’ hopes and fears for the future.

An example of informal climate education is the Museum of Climate Hope.^1^ A museum trail across seven different institutions was created with supplemental digital content. The museum objects–and species at the garden–were chosen by students, educators, curators and researchers to explore the themes of resilience, innovation and transformation. This educational experience illustrates the handrail of awareness (of self and world) and perspectives lens looking at interdisciplinarity, with institutions and their collections covering humanities, social sciences, and STEM subjects. The Museum of Climate Hope also applied the justice lens by inviting acknowledgment of historic injustices, for example the items in the anthropology and archeology museum include a Hawaiian feather cloak (sustainably harvested from now endangered and extinct species) that was given as a gift to a representative of the British Empire, as well as a reindeer parka from the Evenki people in Siberia. In both cases, themes such as indigenous stewardship of place and the legacies of colonization inform modern understandings of climate vulnerability engaging the perspective and empathy lenses.

As a relatively small-scale pilot engagement project, the Museum of Climate Hope is not presented as proof of the Hope Wheel, but rather as an example of how the components of this model were incorporated into program design and delivery. Future research and evaluation activities are required to explore in more detail the relationship between such interventions and measurements of self-reported climate hope.

Through the relationships developed with schools in the above projects, the Museum of Climate Hope team was invited to deliver an assembly on climate hope to the sixth form (ages 16 to 18) of a local secondary school. This invitation followed a presentation to the same students by a climate scientist who used the opportunity to focus on the hard facts of climate catastrophe, illustrated by visuals of destruction and suffering. Afterward, students complained that the climate scientist misread their needs, and, in trying to wake them up to the climate crisis, only deepened levels of disengagement and despair. In this light, honesty about the situation needs to be balanced with honesty about possible solutions, while at the same time creating safe/brave spaces that lead to increased self-awareness and empower action.

#### 4.1.2 Awareness and action handrails operationalized

Operationalizing the handrails of self-awareness and honesty in the Hope Wheel, d’Abreu (2022a,b) used Freire’s praxis-based pedagogy to engage students to envision change needed around social, economic and environmental challenges. Students were invited to apply theories of culture and communication–for example, Tajfel’s Social Identity Theory (Tajfel and Turner, 1979) and Hall (1989) Cultural Iceberg model (1989)–to their own lived experience to identify issues of prejudice, stereotyping and othering they perceived within their cultural landscapes. Students created a video entitled “My Cultural Identity,” in which they reflected on and identified problematic identity representations within cultures, and suggested ways in which these might be challenged and changed. This invited reflection on self and world identities and envisioning possible solutions to the situation with the aim of enabling hope through the learning process. A “cinema screening” of videos was shown in plenary at the end of the course, collectively sharing these multiple perspectives and communicating possible actions to the issues students identified.

A further example is students in Oxford, UK collaborating with students in Grenoble, France on a COIL project (Co-operative, Online International Learning) in which they researched an issue of social, economic or environmental significance, comparing its causes, impacts and related campaigns in their distinct geographical locations. They conducted research and compared potential individual actions and collective responses locally and shared these with their team members via an online noticeboard. Here honesty around the situation and solutions was required and awareness of self and world was developed. Students worked on topic areas such as ecocide and fast fashion, plastic pollution and food poverty, generating possible solutions and pathways working with the aforementioned guardrails and engaging the lenses of complexity, justice, perspectives and creativity.

### 4.2 Climate hope pitfalls

This model builds on the premise that active, constructive, and transformative conceptualizations of hope provide a means of purposefully engaging climate change learning. However, it is worth briefly reflecting on more critical approaches to the concept of hope in the context of climate change.

One critique of climate hope could be characterized as imposed or outsourced hope, in which the burden of hope is imposed on others, versus participating in individual and collective action. This imposition of hope was described by Bill McKibben during an interview with climate activist Xiye Bastida: ‘When they say, “You give me hope,” part of what they’re saying is, “I don’t want to feel so bad about myself”’ (Schwartz, 2023).

As captured in the guardrail of false hope, there can be conceptualizations of hope that are disempowering and unproductive. The observations above of outsourcing hope by imposing it on young climate activists raises the question of whose hope, and to what end? When used to justify an avoidance of discomfort and lack of action, this form of hope is not a productive form of engagement with climate change. This sentiment was echoed by Greta Thunberg when she noted that “hope is not passive, hope is not blah, blah, blah…hope is telling the truth and taking action” (Thunberg, 2021).

The Hope Wheel provides a constructive framework for engaging with hope-based pedagogies. Without being prescriptive, the handrails, guardrails and lenses signpost key practical elements and considerations for educators to address in CCE while also identifying some of the potential pitfalls around unhelpful climate hope narratives.

As mentioned above, this Curriculum, Instruction and Pedagogy article is not a systematic review or original research, but rather contribution to the environmental and sustainability education community building on research and practice in educational psychology related to climate change and hope. Researchers and practitioners are invited to apply, adapt and critique the Hope Wheel, with future research necessary to validate the efficacy of this model.

## 5 Conclusion

*Hope doesn’t soothe pain*

*with pleasantries but is a tender*

*reminder that the door to transformation*

*is always open (Nwulu, 2023)^2^*

A wheel presents a tool with structural integrity due to the intersection of the hub, spokes and rim. We use wheels to travel forward with a desired end destination in mind. The Hope Wheel aims to simplify core tenets in the literature on hope in CCE to support educators. In particular, this model offers guidance on what to include (handrails), what to avoid (guardrails) and important considerations (lenses) when designing and implementing formal and informal learning experiences.

It affirms that educators can create spaces for difficult conversations while protecting learner well-being, support honest explorations of hard climate change truths while addressing misconceptions, and facilitate the journey of self-awareness toward individual and collective action. There is no single model for pedagogies of hope in CCE, and the applications outlined above illustrate some examples of different approaches to implementing these concepts in practice. Educators are invited to reflect on aspects of the wheel they are already applying in their teaching practice and to explore how these might be enhanced or developed.

Learning about climate change can be uncomfortable, but in these moments of discomfort are the seeds of transformation. As educators, we do not need to have all the answers–armed with creativity and care, mindful of different perspectives and climate justice, we can all cultivate hope and equip learners with tools to navigate this time of change and uncertainty.

## Author contributions

WF: Writing—original draft, Writing—review and editing. Cd: Writing—original draft, Writing—review and editing.

## Data Availability Statement

The original contributions presented in the study are included in the article/supplementary material, further inquiries can be directed to the corresponding author/s.

## Footnotes

1 The Museum of Climate Hope is a partnership between the Environmental Change Institute (ECI) and the Gardens, Libraries and Museums (GLAM) at the University of Oxford with funding from the Public and Community Engagement with Research seed fund. More information is available at http://climatehope.uk/.

2 Excerpt from “Like Prayer” by Selina Nwulu, a poem presented at the *Hope and Action* panel at the Everything is Connected season of the Cultural Programme, Humanities Division, University of Oxford, 28 October 2023.
